# scRNA+TCR-seq reveals the pivotal role of dual receptor T lymphocytes in the pathogenesis of Kawasaki disease and during IVIG treatment

**DOI:** 10.3389/fimmu.2024.1457687

**Published:** 2024-10-03

**Authors:** Yuanyuan Xu, Yi Yuan, Lanlan Mou, Linhu Hui, Xing Zhang, Xinsheng Yao, Jun Li

**Affiliations:** ^1^ Department of Immunology, Center of Immunomolecular Engineering, Innovation & Practice Base for Graduate Students Education, Zunyi Medical University, Zunyi, China; ^2^ Department of Cardiology, Kunming Children’s Hospital, Kunming, China

**Keywords:** dual TCR T cell, Kawasaki disease, IVIG treatment, Treg, CDR3

## Abstract

**Introduction:**

Kawasaki disease (KD), a common cause of acquired heart disease in children in developed countries, is primarily treated with intravenous immunoglobulin (IVIG), but some children demonstrate IVIG resistance with increased coronary artery injury risk. T cells have been demonstrated to be involved in the pathogenesis of KD and its treatment with IVIG. However, the role and mechanism of dual TCR T lymphocytes in the occurrence of KD and IVIG therapy remain unclear.

**Methods:**

This study, based on scRNA-seq combined with TCR-seq technology, clustered the peripheral blood mononuclear cells of 3 healthy controls and 6 KD patients before and after IVIG treatment. Comparative analysis was conducted to investigate the differences in the proportion of single/dual receptor T cells, the characteristics of CDR3 repertoires, cell types, and the expression of transcription factors among the three groups. The study aimed to explore the correlation between dual TCR T cells and KD as well as IVIG treatment.

**Results:**

In our experimental results, we observed the presence of dual TCR T cells in all three groups. However, compared to the healthy control group and the IVIG-treated group, the KD patients before IVIG treatment exhibited a lower proportion of dual TCR T cells, with variability between samples, ranging from 4% to 15%. Notably, after IVIG treatment, the proportion of dual TCR T cells significantly increased, stabilizing above 12%, and these T cells also exhibited clonal expansion and a preference for V gene usage. In addition we found differences in dual TCR T cell subsets among the three groups, for example, IVIG treatment increases the proportion of dual TCR Treg cells, but it still remains below that of healthy control groups, significantly higher proportions of both dual TCR CD8 central and effector memory T cells in IVIG-treated KD patients, and differences in the expression of transcription factors between single and dual TCR T cells. These results suggest dual TCR T cells correlate with KD and IVIG treatment.

**Conclusion:**

Dual TCR T lymphocytes, especially dual TCR CD8 T cells and Treg cells, play crucial roles in the pathogenesis of KD and during IVIG treatment, providing strong support for further elucidating KD pathogenesis and optimizing treatment strategies.

## Introduction

Kawasaki disease (KD) is a systemic vasculitis predominantly affecting infants and young children under the age of 5, characterized by bilateral conjunctival injection, polymorphous skin rash, changes in oral mucosa, and non-purulent lymphadenopathy (1). KD can lead to severe cardiovascular complications such as coronary artery dilatation or aneurysm during the acute phase and has replaced rheumatic fever as a major cause of acquired heart disease in developed countries. The pathogenesis of KD involves various factors including infection (2), genetic susceptibility (3), congenital and adaptive immune abnormalities (4, 5). Current treatment for KD relies mainly on intravenous immunoglobulin (IVIG) infusion and high-dose aspirin, which reduces the risk of coronary artery aneurysms (CAA) to 3-6% in all treated children (6). However, approximately 10-15% of patients do not respond positively to this treatment (7).

Although the exact mechanisms driving KD pathogenesis and IVIG treatment are still unclear, comprehensive research has shown that T lymphocytes play a crucial role in both KD pathogenesis and the effectiveness of IVIG therapy. Systemic vasculitis is a hallmark clinical feature of KD, with Brown et al. identifying a substantial involvement of CD8+ T lymphocytes in vascular lesions of KD patients (8). During the acute phase of KD, there is an imbalance in T lymphocyte subset networks, characterized by altered proportions of CD4+ and CD8+ subsets, reduced proportions and functional defects in Treg cells (9), and increased CD40L expression on T cell surfaces (10). IVIG treatment enhances regulatory T cell activity, suppresses inflammatory cytokine production (11), and balances Th1/Th2 immune responses (12). Investigating the pathogenic T cell subsets in KD is of paramount importance for a deeper understanding of the immunopathological mechanisms of KD and the development of more effective therapeutic strategies.

The clonal selection theory has long been considered fundamental to T cell development, tolerance selection, and specific responses (13). This theory posits that a lymphocytes only express one TCR or Ig chain from the same allele, so as to ensure the recognition of a single specific antigen, which is beneficial for the central tolerance checkpoint to clear the self-reactive lymphocytes, thus inhibiting autoimmune responses (14, 15). However, some studies have reported the phenomenon of individual T cells expressing two different TCRs. In 1988, functional Vα and Vβ gene rearrangements were detected at the mRNA level in single-clonal T cells (16, 17). Subsequently, many labs confirmed incomplete V(D)J allelic exclusion of TR chains and identified dual TCR T cells in both central and peripheral immune organs of humans and mice (18–20). Given the disruptive advantage of scRNA+TCR-seq technology in analyzing TCR pairing, we also identified a high proportion of dual TCR T cells in human and murine thymus and peripheral lymphoid organs (21), with a significant increase in the proportion of dual TCR T cells in the elderly population (22).

In recent years, the involvement of dual-receptor lymphocytes in regulating autoimmune diseases has been established. Bradley et al. (23) found that segmented filamentous bacteria induced autoimmune arthritis by remotely modulating Th17 cell responses via the gut-lung axis while selectively expanding dual-receptor Th17 cells. In type 1 diabetes, lymphocytes with both TCR and BCR expression (DEs) have also been discovered (24). Clonally expanded DEs encode an effective self-antigen in the variable region of the Ig heavy chain, carrying monoclonal antibodies reactive to CD4 T cells and inhibiting the binding of insulin tetramers to CD4 T cells. Building on the disruptive advantage of scRNA+TCR-seq technology in TCR pairing analysis, this study identified for the first time dual TCR T cells in KD patients and observed significant changes in their proportions after IVIG treatment, suggesting their potential involvement in the autoimmune inflammatory response of KD patients and the effects following after IVIG treatment.

## Materials and methods

### Sample information

**Figure 1:**
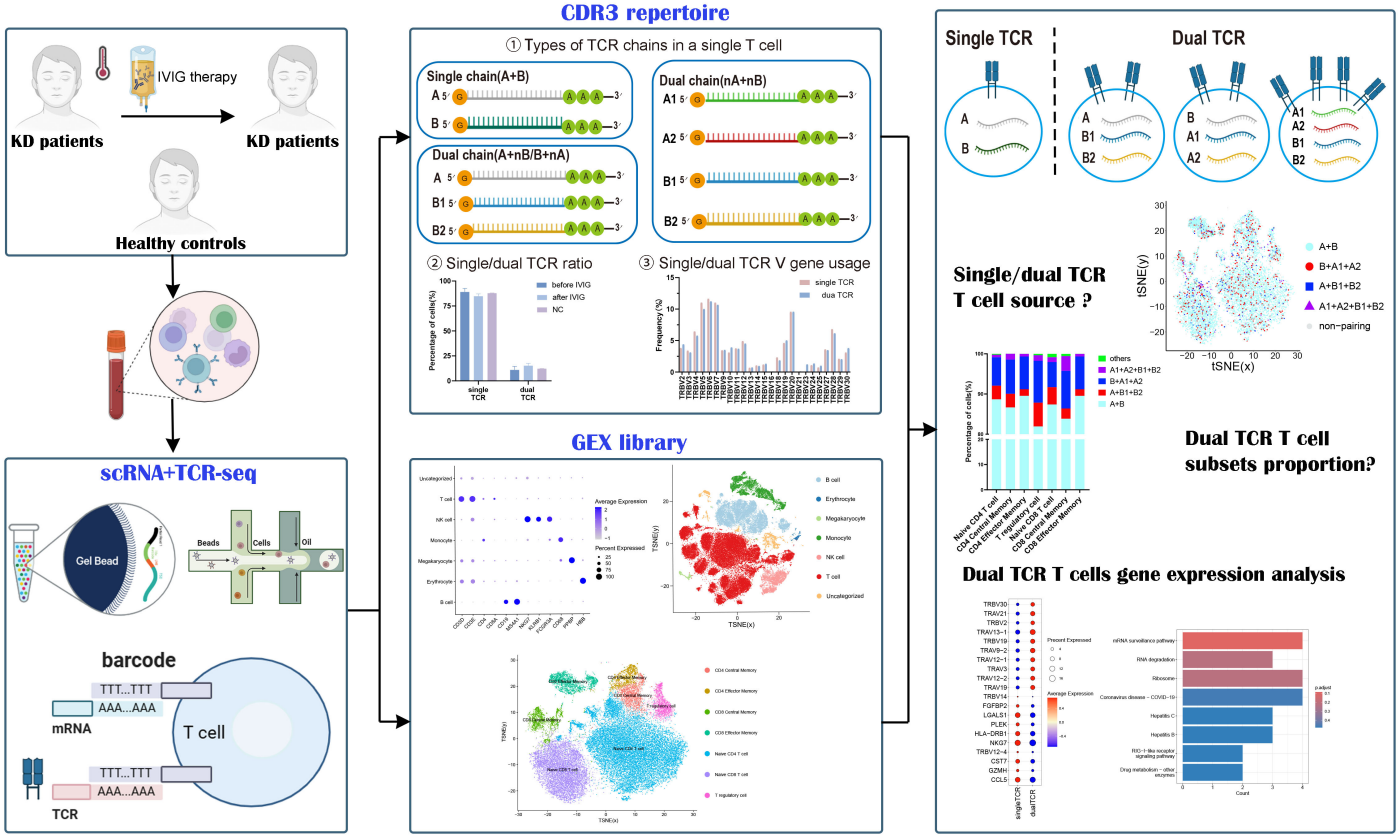
Experimental workflow for characteristics analysis of single/dual TCR cells.

This study utilized single-cell RNA sequencing (scRNA-seq) and T cell receptor sequencing (TCR-seq) data from *the Gene Expression Omnibus (GEO) data repository* (accession number: *GSE168732*) for analysis. The dataset comprised samples from KD patients and healthy controls, totaling 15 samples, including 3 healthy volunteers and 6 volunteers each from KD patients before and after IVIG treatment. Detailed research methodology was shown in **Figure 1**. Inclusion criteria for patients with KD(age: 1.6-5.4 years) According to the diagnostic guidelines for Kawasaki disease formulated by the American Heart Association in 2017 (6): Fever of unknown etiology lasting for ≥5 days、conjunctivitis、oral changes、limb changes、rash and cervical lymphadenopathy, if at least four symptoms are met, it is diagnosed as complete Kawasaki disease, otherwise it is incomplete Kawasaki disease, all patients were treated with high-dose IVIG(1 g/kg per day) combined with oral aspirin (30 mg/kg per day) after the diagnosis of KD. Healthy control group with similar age (age: 1.2–5.5 years) comes from routine physical examination and has no fever recently, infection or immunization. These 15 samples were categorized into three groups for further analysis: The pre-IVIG treatment group (6 KD patient samples), the post-IVIG treatment group (6 KDpatient samples), and the healthy control group (3 healthy individual samples).

### Data quality control

The obtained scTCR-seq data from the 15 samples has been successfully annotated with sequences. Based on the annotated data, we further processed the data by eliminating sequences that did not meet specific criteria. This included removing sequences marked “FALSE” in the “is_cell” and “high_confidence” columns, excluding sequences in the “chain” column that were not “TRA” or “TRB,” eliminating sequences labeled as “Non” or “FALSE” in the “productive” column, and excluding cells with single functional chains.

The scRNA-seq data from the 15 samples were analyzed using R language (version 4.2.3) and Seurat package (version 4.4.0). Sample quality control was performed based on the following criteria: total gene count > 500, UMI count between 1000 and 20000, and mitochondrial gene percentage < 5%. Moreover, the DoubletFinder R package (version 2.0.4) was used to identify doublet cells in each sample, which were subsequently removed.

### scRNA-seq data processing and analysis

Dimensionality reduction and clustering analysis of the scRNA-seq data were performed after quality control. The t-SNE method was applied to reduce data dimensionality, selecting the top 30 principal components for subsequent analysis. Subsequently, the K-means clustering algorithm was utilized to cluster the data after dimensionality reduction, resulting in 15 cell clusters. T cells were identified and filtered from the entire cell population based on specific markers or gene expression patterns. Seurat package was then employed to further identify and classify T cell subsets. Lastly, cell clusters were annotated using classical gene expression analysis and the SingleR package (version 2.0.0), with the cell clusters and gene expression profiles presented in **Supplementary Figure 1**.

### Discrimination of single and dual TCR T cell

Using Excel, single TCR T cells (cells with one type of α chain and one type of β chain) and dual TCR T cells (cells with additional functional α or β chains beyond single TCR T cells) were filtered in each sample based on individual α and β chain V(D)J gene sequences. Subsequently, the numbers of single TCR and dual TCR paired T cells were analyzed for each sample, and the proportions of various TCR pairing types were calculated. Details of single TCR and dual TCR paired cells are provided in **Supplementary Table 2**.

### Analysis of dual TCR T cell characteristics

T cells were categorized into 7 subgroups: Naive CD4 T cell, Naive CD8 T cell, CD4 central memory, CD8 central memory, CD4 effector memory, CD8 effector memory, and Treg. Subsequently, TCR information was added to Seurat objects using the AddMetaData function to determine the origin of single and dual TCR T cells in the three groups. The proportions of single TCR and dual TCR T cells in each cell subgroup were calculated, and differential expression analysis of the top 10 mRNA molecules between single TCR and dual TCR T cells within each T cell subgroup was performed using DESeq2 R package. Additionally, CDR3 clonality and V/J gene usage analysis was conducted on single/dual TCR T cells using the immunarch package in R.

### Statistical analysis

Data processing and statistical analyses were performed using R language and IBM SPSS Statistics 18 software. Independent sample t-test was used for continuous variable comparisons between two groups, while chi-square test was used for categorical variable comparisons. Paired sample t-tests (paired t-test) were employed to investigate changes in single TCR and dual TCR T cell proportions in KD patients before and after IVIG treatment. For comparisons among multiple groups, analysis of variance (ANOVA) was utilized. p-values < 0.05 were considered statistically significant.

## Results

### The ratio of single to dual TCR T cells

In accordance with their receptor composition, T cells are classified into two types: αβ T cells and γδ T cells. This study exclusively focuses on αβ T cells. After quality control, the cell counts of 6 patients with KD before and after IVIG treatment, as well as 3 healthy controls, ranged from 1707 to 6352. Among these, the numbers of T cells capable of simultaneously detecting α and β chains within a single cell ranged from 1217 to 5647.

**Figure 2:**
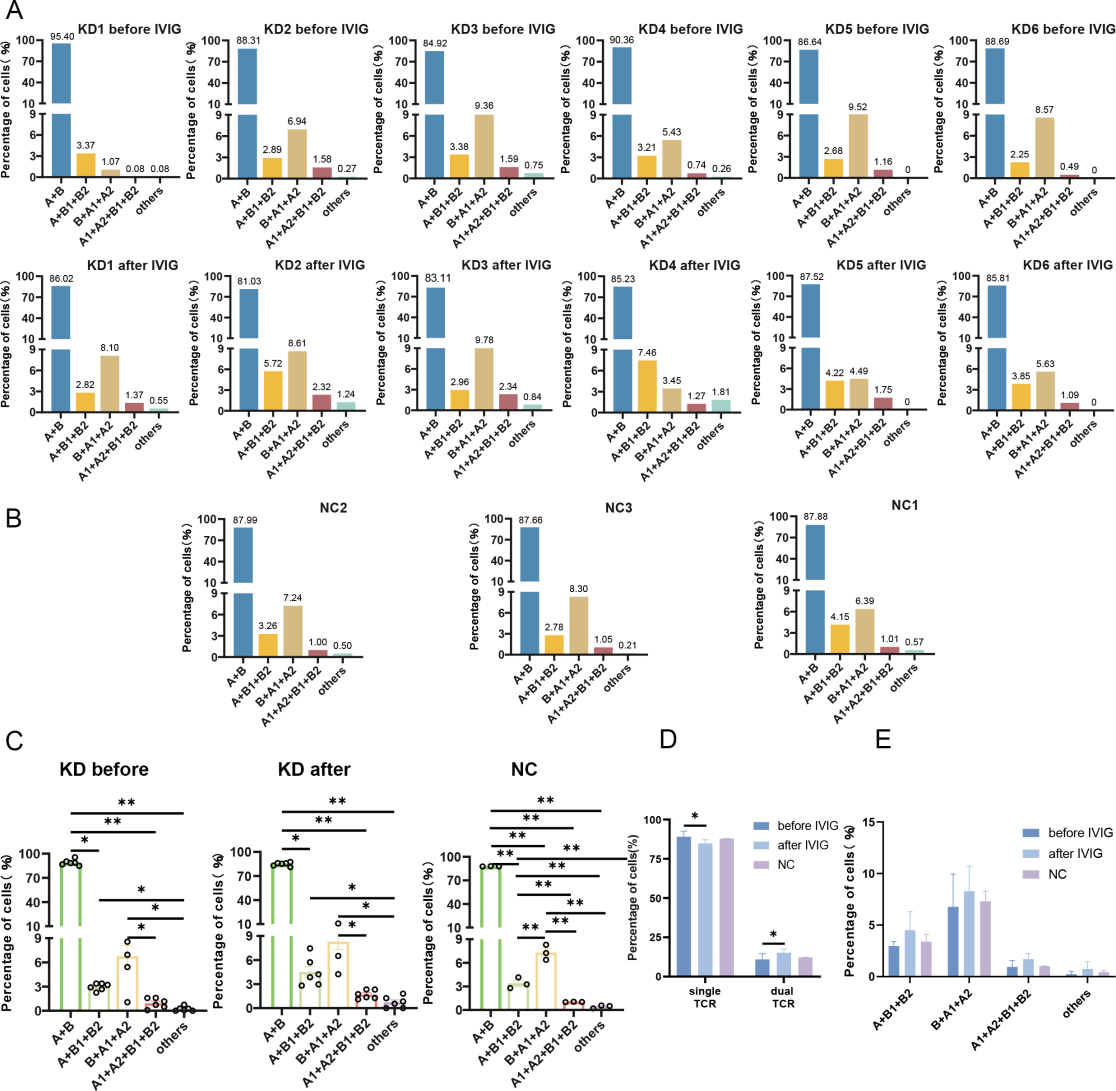
Proportions of single and dual TCR T cells. **(A)** Proportions and types of single TCR T cells and dual TCR T cells in 6 KD patients before and after IVIG treatment; **(B)** Proportions and types of single TCR T cells and dual TCR T cells in 3 healthy control subjects; **(C)** Statistical analysis of 5 types of dual TCR T cells in each group; **(D)** Comparative statistical analysis of single/dual TCR T cells among the three groups; **(E)** The proportions of the different subtypes of dual TCR T cells across the three groups. KD, Kawasaki disease; IVIG, intravenous immunoglobulin; **(A)** α chain; **(B)** β chain. *P<0.05; **P<0.01.

In each sample, T cells were predominantly single TCR T cells (over 85%), but a certain proportion of dual TCR T cells (approximately 15%) were present in all 15 samples (**Table 1**). It is noteworthy that, with the exception of sample 5 from the KD group, the proportion of dual TCR T cells in patients with KD increased after IVIG treatment compared to their pre-treatment levels. In patients KD1 and KD3, an increase in dual TCR T cells of the B+A1+A2 subtype was predominant. In samples from KD4 and KD6, an increase in dual TCR T cells of the A+B1+B2 subtype was predominant. In KD2 samples, all four types of dual TCR T cells showed an increased proportion (**Figure 2A**). All four types of dual TCR T cells were detected in the healthy control group (**Figure 2B**). The predominant pairing type of dual TCR T cells across the three groups of samples was B+A1+A2 (**Figure 2C**).

**Table 1:** The proportions and types of single/dual receptor T cells in all samples.

Sample	group	GEO Accession Number	Total cells/paired TCR cells	Single TCR T cells(A+B)/proportion%	Dual TCR T cells/proportion%				
					Total	A+B1+B2	B+A1+A2	A1+A2+B1+B2	others
KD1	IVIG before	GSM5160417	2354/1217	1161/95.40	56/4.60	41/3.37	13/1.07	1/0.08	1/0.08
KD2	IVIG before	GSM5160420	3283/2593	2290/88.31	303/11.68	75/2.89	180/6.94	41/1.58	7/0.27
KD3	IVIG before	GSM5160422	3539/3076	2612/84.92	464/15.08	104/3.38	288/9.36	49/1.59	23/0.75
KD4	IVIG before	GSM5160424	3284/2302	2080/90.36	222/9.64	74/3.21	125/5.43	17/0.74	6/0.26
KD5	IVIG before	GSM5160427	3276/2837	2458/86.64	379/13.35	76/2.68	270/9.52	33/1.16	0/0.00
KD6	IVIG before	GSM5160430	1707/1424	1263/88.69	161/11.31	32/2.25	122/8.57	7/0.49	0/0.00
KD1	IVIG after	GSM5160419	3322/2911	2504/86.02	407/13.98	82/2.82	269/9.24	40/1.37	16/0.55
KD2	IVIG after	GSM5160421	6352/5647	4576/81.03	1071/18.97	323/5.72	547/9.69	131/2.32	70/1.24
KD3	IVIG after	GSM5160423	4835/4398	3655/83.11	743/16.89	130/2.96	473/10.75	103/2.34	37/0.84
KD4	IVIG after	GSM5160425	3187/2600	2216/85.23	384/14.77	194/7.46	110/4.23	33/1.27	47/1.81
KD5	IVIG after	GSM5160428	4235/2916	2552/87.52	364/12.48	123/4.22	190/6.52	51/1.75	0/0.00
KD6	IVIG after	GSM5160431	3163/1924	1651/85.81	273/14.19	74/3.85	178/9.25	21/1.09	0/0.00
NC1	NC	GSM5160432	4087/3350	2944/87.88	406/12.12	139/4.15	214/6.39	34/1.01	19/0.57
NC2	NC	GSM5160434	3016/2389	2102/87.99	287/12.01	78/3.26	173/7.24	24/1.00	12/0.50
NC3	NC	GSM5160435	3285/2844	2493/87.66	351/12.34	79/2.78	236/8.30	30/1.05	6/0.21

KD, Kawasaki disease; IVIG, intravenous immunoglobulin; GEO, gene expression omnibus; A, α chain; B, β chain

The four types of dual TCR T cells were combined, and the difference in the ratio of single TCR to dual TCR T cells between the three groups was calculated. It was found that after IVIG treatment, the proportion of single TCR T cells significantly decreased compared to pre-treatment levels, while the proportion of dual TCR T cells significantly increased. However, there was no statistically significant difference observed between the patient groups and the healthy control group (**Figure 2D**). Although the overall proportions of the different dual TCR T cell subsets did not show significant differences among the three groups, there was an increase in all four subsets of dual TCR T cells in patients after IVIG treatment compared to before treatment (**Figure 2E**).

### The repertoire characteristics of the dual TCR T cells

**Figure 3:**
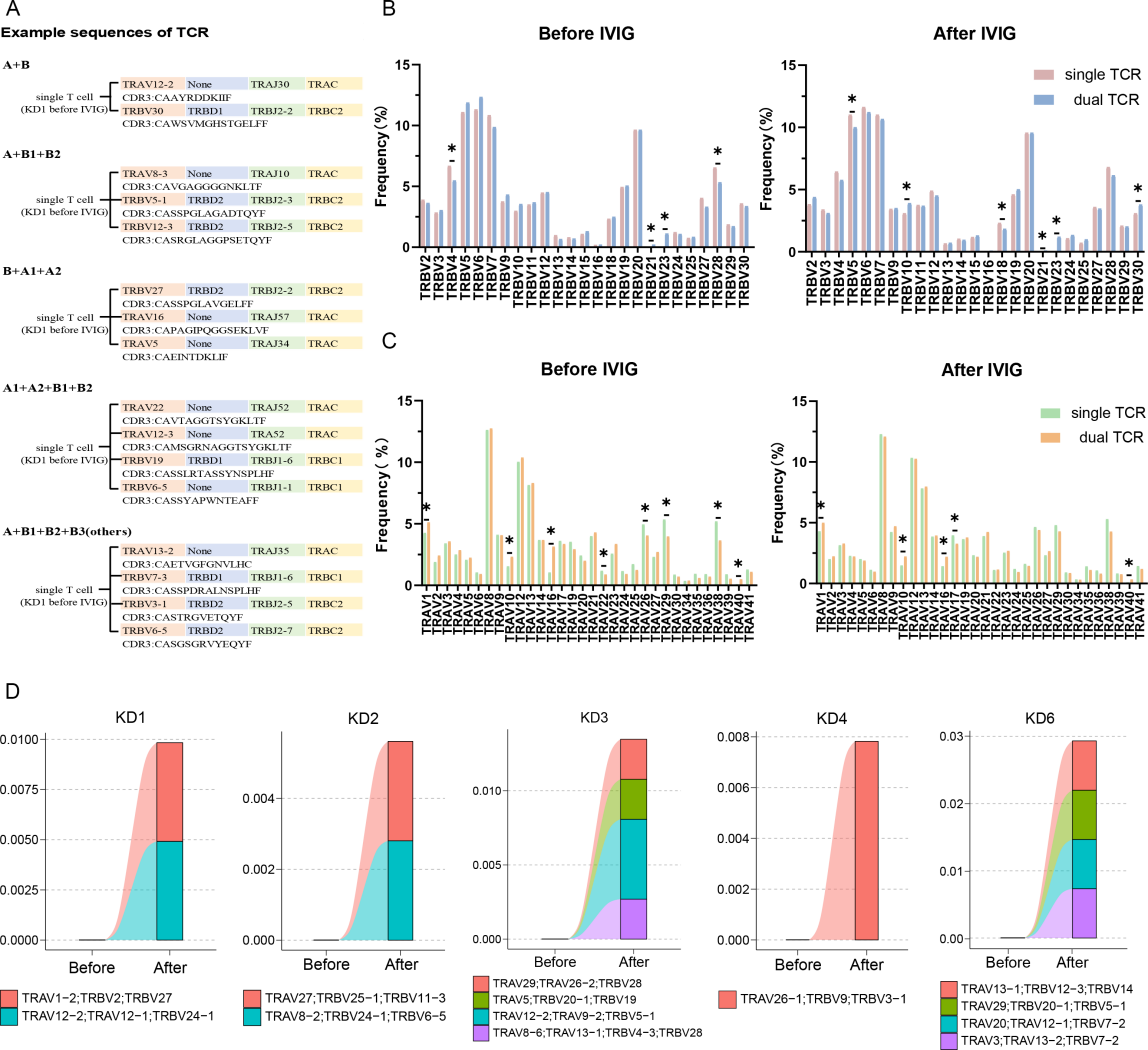
Characteristics of CDR3 region sequences in single and dual receptor T cells. **(A)** Schematic representation of single T cell expressing different TCR α and β chains; **(B)** Usage of TRBV genes in single and dual TCR T cells before and after IVIG treatment; **(C)** Usage of TRAV genes in single and dual TCR T cells before and after IVIG treatment; **(D)** Clonal expansion of dual TCR T cells after IVIG treatment. KD, Kawasaki disease; IVIG, intravenous immunoglobulin; **(A)** α chain; **(B)** β chain. *P<0.05.

In 15 samples of T cells, simultaneous expression of two or more α chains or β chains was observed. (**Figure 3A**) illustrated an example of TCR T cell VDJC composition in KD1 sample before treatment, confirming the presence of allelic gene rearrangements in both α and β chains of T cells. Both single and dual TCR T cells showed biased usage of TRBV and TRAV genes before and after treatment. However, before and after treatment, dual TCR T cells frequently utilized *TRBV21*, *TRBV23*, *TRAV1*, *TRAV10*, *TRAV16*, and *TRAV40* genes. Additionally, significant increases in gene usage of *TRBV10* and *TRBV30* in dual TCR T cells were observed in samples after IVIG treatment (**Figures 3B, C**). Upon observing the characteristics of changes in dual TCR T cells before and after treatment, we defined clonal expansion as clonal size ≥2. It was observed that except for the KD5 sample, all other samples exhibited clonal expansion of dual TCR T cells after IVIG treatment (**Figure 3D**). Detailed information on clonal expansion sequences and pre- and post-treatment proportions can be found in **Supplementary Table 1**.

### The distribution of single and dual TCR T cells in different subsets

**Figure 4:**
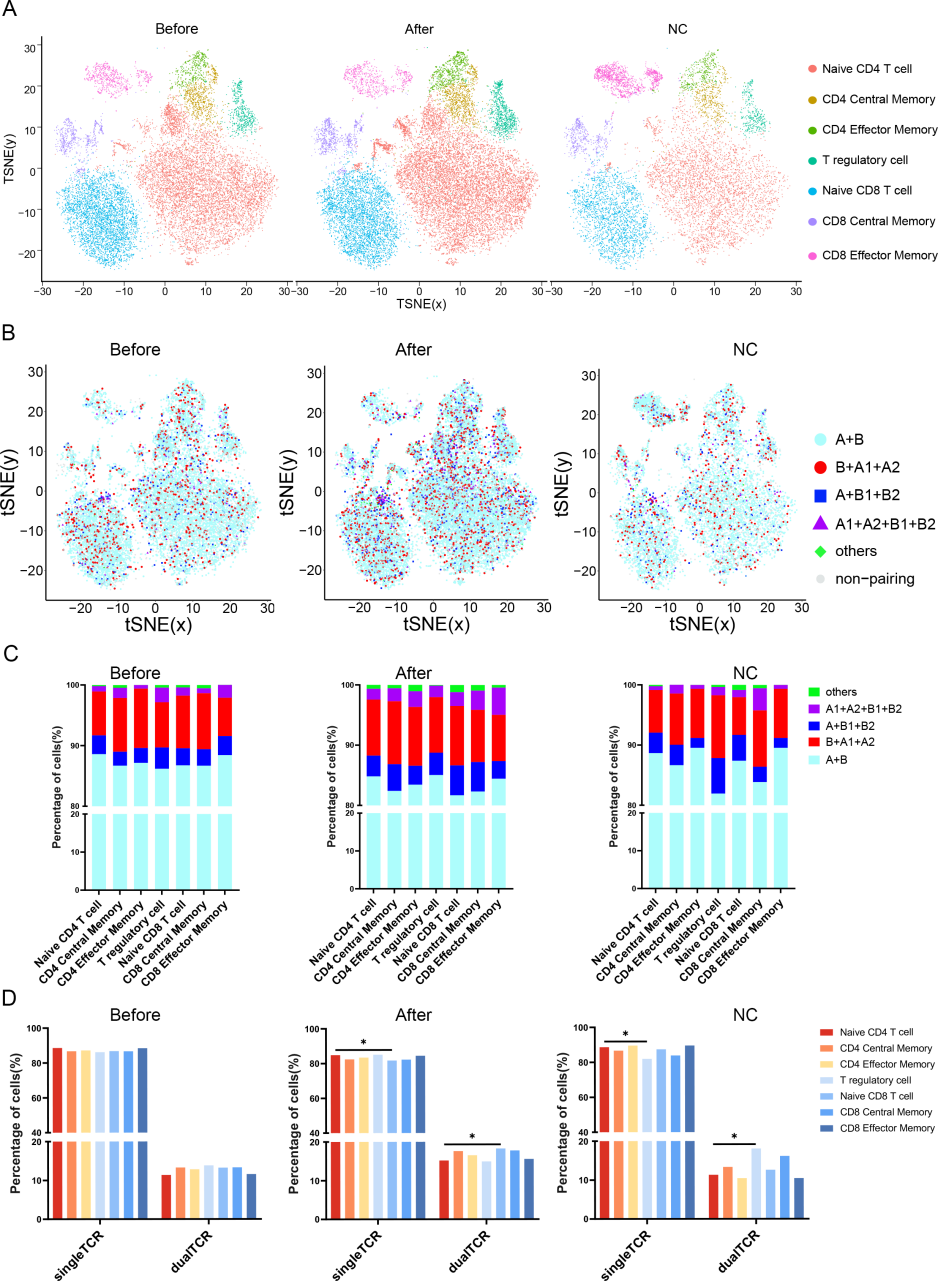
Distribution and proportions of single and dual receptor T cells across different subsets. **(A)** The UMAP plot illustrates the distribution of 7 T cell subpopulations before VIG treatment, after IVIG treatment, and in the control group; **(B)** The figure depicts the distribution of 5 types of TCR T cells before and after IVIG treatment, as well as in the control group; **(C)** This figure demonstrates the proportions of 5 types of TCR T cells before IVIG treatment, after IVIG treatment, and in the control group; **(D)** Statistical analysis reveals the proportions of single and dual TCR T cells before VIG treatment, after IVIG treatment, and in the control group. *P<0.05.

After dimensionality reduction clustering of the 15 samples, cell populations were annotated based on classical gene expression(**Supplementary Figure 1**). The cell subsets and gene expression levels for each are detailed in **Supplementary Figure 2**. Seven cell subsets were identified among the three groups (**Figure 4A**), with each subset in the three groups containing both single TCR T cells and four types of dual TCR T cells (**Figure 4B**). Single TCR T cells were the predominant cell type in all three groups, while each sample subgroup expressed the four types of dual TCR T cells, with proportions around 15%, primarily of the B+A1+A2 type.

Analysis of the source of single/dual TCR T cells revealed that in the healthy control group, dual TCR T cells mainly originated from the Treg subtype, with a significantly higher proportion of dual TCR Treg compared to dual TCR Naive CD4 T cells. Among the seven cell subtypes, the total percentage of dual TCR T cells in KD patients after IVIG treatment increased compared to pre-treatment levels (**Figure 4C**). Post-treatment, the proportion of dual TCR Naive CD8 T cells in patients was significantly higher than that of dual TCR Naive CD4 T cells (**Figure 4D**).

### The transcription differences between single and dual TCR T cells

**Figure 5:**
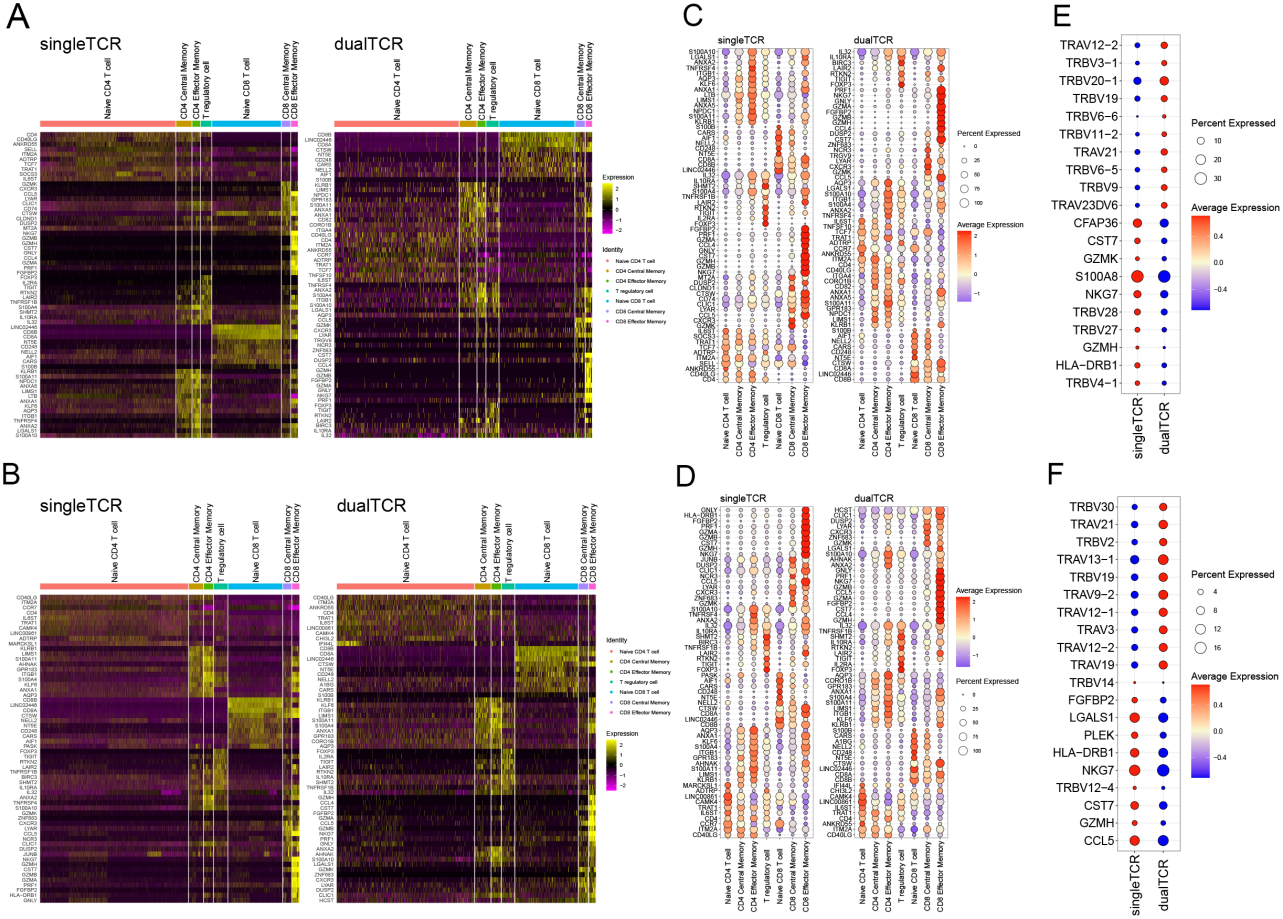
Differential gene expression of single and dual TCR T cells between different subgroups before and after IVIG treatment. **(A)** Heatmap of differential gene expression of single and dual TCRs among different subgroups before treatment. **(B)** Top 10 genes expressed in single and dual TCR T cells among 7 cell subpopulations before treatment. **(C)** Top 10 highly expressed genes in total single TCR T cells and total dual TCR T cells before treatment. **(D)** Heatmap of differential gene expression of single and dual TCRs among different subgroups after treatment. **(E)** Top 10 genes expressed in single and dual TCR T cells among 7 cell subpopulations after treatment. **(F)** Top 10 highly expressed genes in total single TCR T cells and total dual TCR T cells after treatment.

The high-expression genes between single and dual TCR T cells in the 7 T cell subsets of KD patients before and after IVIG treatment are largely consistent (**Figures 5A, B**). Both single and dual TCR T cell subsets exhibit high expression of their respective characteristic genes, such as naive cells (CCR7, TCF7, SELL), central memory cells (CCR7, SELL, S100A4), effector memory cells (S100A4), and Treg (FOXP3, KLRB1) (**Figures 5C, D**). The identification of these subsets validates the accuracy of our analysis.

By analyzing the expression differences between all single and dual TCR T cells before and after treatment, we found that they mainly differ in the usage of TRAV and TRBV subfamilies. Specifically, there is a notable shift in V gene usage among dual TCR T cells pre- and post-treatment. Before treatment, dual TCR T cells showed a higher frequency of TRBV genes, such as TRBV20-1, TRBV19, and TRBV9, with 7 of the top 10 differentially used V genes being TRBV genes (**Figure 5E**). After treatment, dual TCR T cells exhibited a shift towards higher usage of TRAV genes, such as TRAV13-1, TRAV9-2, and TRAV19, with 7 out of the top 10 differentially used V genes being TRAV genes (**Figure 5F**). These changes in V gene usage suggest that IVIG treatment may alter the antigen recognition profile of dual TCR T cells, potentially enhancing their ability to recognize different antigenic epitopes.

### Differential gene expression and functional enrichment in dual TCR CD8 memory cells

**Figure 6:**
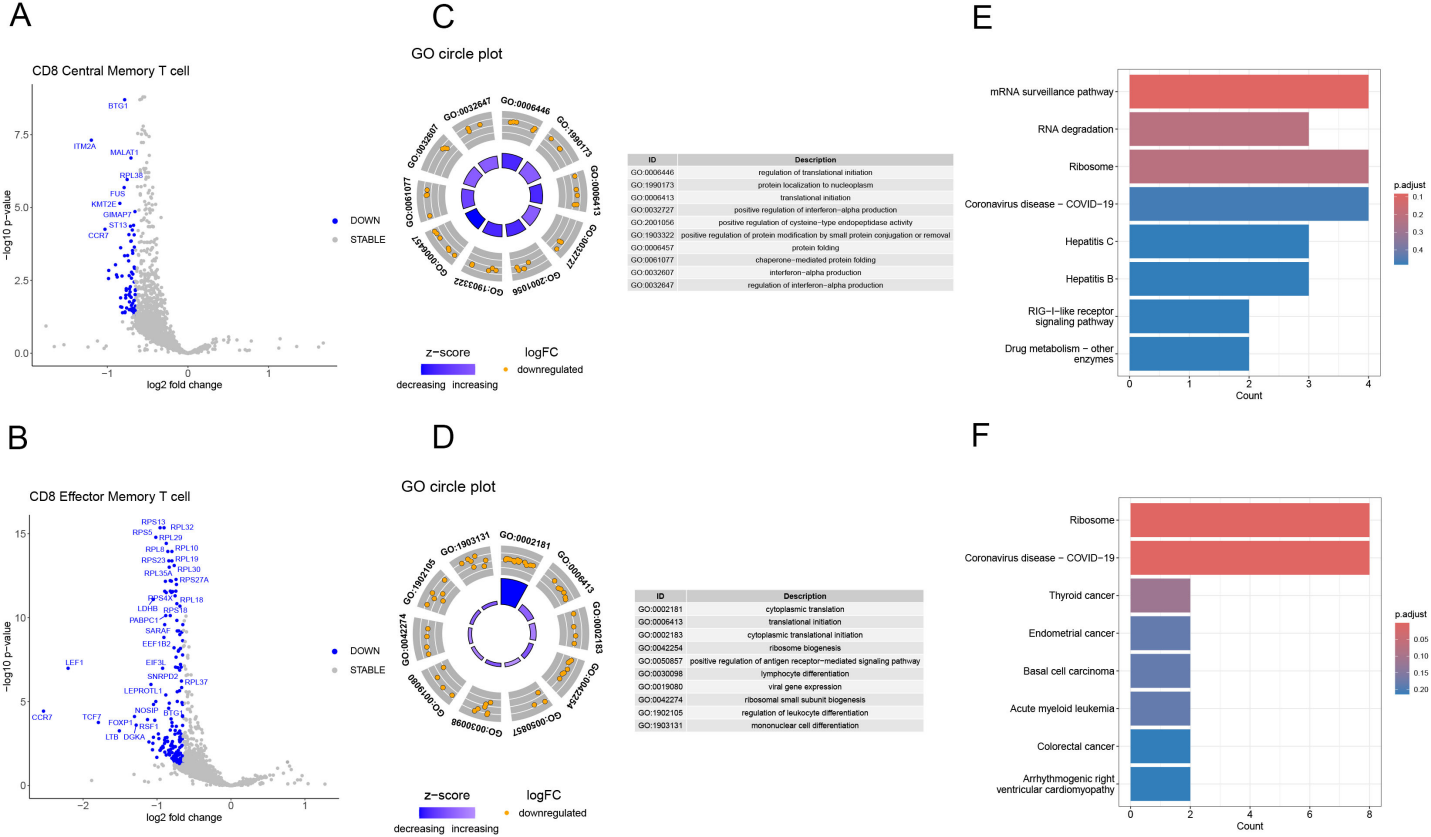
Characterization of gene expression profiles of dual TCR central and effector memory CD8 T cells in IVIG-treated patients. **(A)** Volcano plot depicting differentially expressed genes in dual TCR central memory CD8 T; **(B)** Volcano plot depicting differentially expressed genes in dual TCR effector memory CD8 T; **(C)** GO enrichment analysis of differentially expressed genes in dual TCR central memory CD8 T cells; **(D)** GO enrichment analysis of differentially expressed genes in dual TCR effector memory CD8 T cells; **(E)** KEGG enrichment analysis of differentially expressed genes in dual TCR central memory CD8 T cells; **(F)** KEGG enrichment analysis of differentially expressed genes in dual TCR effector memory CD8 T cells.

After analyzing the proportions of dual TCR T cells in KD patients before and after IVIG treatment, we observed a significant increase in the proportions of dual TCR CD8 central memory and CD8 effector memory T cells in post-treatment patients(**Supplementary Figure 3**). Subsequently, using the DESeq2 package for differential gene expression analysis, we conducted differential gene expression analysis on single/dual TCR CD8 central memory and effector memory cells in post-treatment patients, identifying 72 and 167 significantly upregulated genes respectively, compared to single receptor cells (**Figures 6A, B**).

For dual TCR CD8 central memory T cells, the differentially expressed genes (DEGs) were significantly enriched in GO terms associated with the regulation of interferon-alpha production, translation initiation, and protein folding. Additionally, the enriched KEGG pathways included virus infection, mRNA surveillance pathway, and RNA degradation (**Figures 6C, D**). On the other hand, dual TCR CD8 effector memory T cells exhibited DEGs enriched in GO terms linked to cytoplasmic translation, lymphocyte differentiation, and positive regulation of the antigen receptor-mediated signaling pathway. The enriched KEGG pathways for these cells included virus infection and Ribosome-related pathways (**Figures 6E, F**).

## Discussion

Dual TCR T cells have a long history of research and specific experimental data supporting their role as natural products of cell differentiation and development. The proposition of this theory has also sparked reevaluation of the classic clone selection theory. With the widespread application of technologies like single-cell sequencing in TCR sequence research (25, 26), there is increasing attention on the proportions and functional studies of lymphocytes expressing single or dual receptors. Our research methodology can distinguish and identify single TCR and dual TCR T cells from a complete T cell receptor repertoire, allowing us to identify the pairing types, origins, and explore the potential functions of dual TCR T cells at the single-cell level.

This study identified dual TCR T cells ranging from 4.6% to 18.97% in KD patients and the healthy control group. The proportion of dual TCR T cells in the healthy control group was consistently around 12%, which is higher than the traditional estimates of 1%-10% reported in earlier studies (20, 27). This discrepancy may be attributed to the low throughput of detection methods used in previous studies, which may not have fully reflected the pairing status of the TCR repertoire.

Our analysis of TCR pairing in dual TCR T cells,revealed that regardless of the patient group (KD before or after treatment);or in healthy controls, the predominant pairing type of dual TCR T cells was β+α1+α2,consistent with our previous research. This suggests that the majority of TCR β chains follow allelic exclusion rearrangement (28), meaning that after successful rearrangement of the “V-D-J” genes of the TCR β chain on one chromosome, further rearrangements of V-D-J genes on the other chromosome are suppressed. Only when the first V-D-J rearrangement fails will the second rearrangement be initiated, ultimately resulting in a T cell expressing only one functional β chain. However, TCR α chains are not affected by allelic exclusion (29), following allelic inclusion rearrangement, meaning that V-J rearrangement of the α chain can occur simultaneously on two alleles, and the α chain locus can undergo multiple progressive rearrangements on the same allele. These reasons collectively lead to a higher proportion of dual α chains compared to dual β chains, consistent with our previous reports (21).

The proportion of dual TCR T cells revealed a significant increase after IVIG therapy. Additionally, a certain proportion of these cells exhibited clonal expansion after treatment, indicating potential involvement of dual TCR T cells in immune regulatory responses following IVIG treatment. However, the specific regulatory mechanisms require further investigation. Furthermore, after IVIG treatment, dual TCR T cells showed a higher frequency of using genes like *TRBV10* and *TRBV30*. This may be related to the involvement of V genes in recombination mechanisms and tolerance selection in dual-receptor cells, which could also mediate the recognition of different antigenic epitopes. Therefore, in future studies, we will further investigate the potential anti-inflammatory effects of dual TCR T cells with high expression of genes like *TRBV10* and *TRBV30*.

Treg cells play a crucial role in regulating and suppressing immune responses within the immune system. In KD cases involving resistance to IVIG and coronary artery complications, there is evidence of weakened Treg responses (9), which are reversed after IVIG treatment (30). In our study, we also observed this phenomenon, where IVIG treatment can upregulate the transcription factor *FOXP3* associated with Treg cells(**Supplementary Figure 4**). Additionally, we found that after IVIG treatment, the proportion of dual TCR Treg cells increased compared to before treatment, yet it remained lower than that in healthy control groups (**Figure 4D**). These findings shed light on the involvement of dual TCR Treg cells in KD pathogenesis and IVIG therapy. As early as 2006, He et al. observed the presence of a certain number of dual TCR Treg cells, which had an impact on the normal immune system (31). Subsequent studies suggested that dual TCR T cells could limit autoimmune reactions by restricting Treg cell activity (32). However, research on the effects of dual TCR T cells is still lacking. In our study, based on the reduced proportion of dual TCR Treg cells in the disease group and the higher expression of immune inhibitory molecules (*FOXP3*) in dual TCR Treg cells (**Supplementary Figure 4**), we hypothesize that dual TCR Treg cells may exert stronger immunosuppression and anti-inflammatory effects compared to single TCR Treg cells.

Studies have shown that the number and function of CD8 T cells in the immune system of KD patients are abnormal, especially during the acute phase of the disease (33, 34). The activation and proliferation of CD8 T cells may be associated with damage to vascular endothelial cells during the disease development, thereby promoting further development of inflammatory responses. In our study, we observed an increase in dual TCR CD8 T cells after IVIG treatment, with a significantly higher proportion of dual TCR naive CD8 T cells compared to dual TCR naive CD4 T cells, suggesting that IVIG may regulate the proliferation and activation of dual TCR CD8 T cells through immunomodulatory effects, thereby promoting the clearance of pathogens or autoantigens in the body. Furthermore, dual TCR T cells, due to their unique characteristic of expressing two T cell receptors, have the opportunity for secondary recognition of viral antigens (35). Therefore, the increased dual TCR CD8 T cells after IVIG treatment may enhance the immune system’s ability to clear pathogens, thus playing a protective role.

Qing et al. found that monitoring the activation level of CD8 T cells reflects the therapeutic effect of IVIG on KD patients. Their results suggest that IVIG exerts anti-inflammatory effects by inhibiting the activation of CD8+ T cells, and excessive activation levels may lead to IVIG resistance (36). However, in this study, by subdividing CD8 T cells into single and dual receptor T cells, we found a significant increase in the proportions of dual TCR CD8 central memory T cells and dual TCR CD8 effector memory T cells in KD patients after IVIG treatment compared to before treatment. Effector CD8 T cells are responsible for direct killing of infected or abnormal cells, while memory CD8 T cells maintain long-term immune memory and respond rapidly upon re-encountering the same pathogen (37, 38). IVIG treatment may exert its therapeutic effect by activating dual TCR CD8 T cells, which transition to memory dual TCR CD8 T cells after clearing pathogens, thereby enhancing the immune system’s protective capacity in the body. From a therapeutic perspective, IVIG may exert anti-inflammatory effects by suppressing abnormal activation of CD8 T cells, thus alleviating inflammation in KD patients (36, 39). However, we also note the impact of IVIG treatment on CD8 T cell subsets, which may be related to pathogen clearance during IVIG treatment, reconstruction of immune memory, and regulation of the immune system. These mechanisms may work together to strengthen the body’s protection against pathogens.

Another evidence of the involvement of dual TCR CD8 T cells in the IVIG treatment process is that dual TCR CD8 effector memory T cells exhibit significantly higher expression genes such as those belonging to the RPS family, RPL family, LDHB, PABPC1, compared to single TCR CD8 effector memory T cells. These genes are mainly enriched in processes like lymphocyte differentiation, positive regulation of antigen receptor-mediated signaling pathway, viral infection, and inflammation. Evidence suggests that KD patients experience concurrent infections (bacterial, respiratory viruses including coronaviruses, and hepatitis viruses) (40–42), thus the high expression of genes related to viral infection in dual TCR CD8 T cells after IVIG treatment may enhance the immune system’s response to pathogens. Moreover, KD is accompanied by systemic inflammatory vasculitis (43, 44), and the enrichment of genes related to inflammation pathways in dual TCR CD8 T cells after IVIG treatment may reflect the immune system’s role in clearing and repairing inflammation. These genes may participate in regulating inflammatory signaling pathways, promoting the gradual resolution of inflammation and tissue repair.

Overall, this study utilized scRNA-seq combined with TCR-seq technology to comprehensively investigate the changes in single and dual receptor T cells, as well as the CDR3 repertoire characteristics in KD pediatric patients before and after IVIG treatment. The study revealed the impact of IVIG treatment on dual receptor T cell subsets in KD patients, providing important clues for further understanding the pathogenesis of KD and optimizing treatment strategies. Future research can further explore the effects of IVIG treatment on the functionality, antigen recognition, and immune modulation mechanisms of dual receptor T cell subsets, aiming to provide more effective strategies and guidance for clinical monitoring and treatment of KD.

## Conclusion

Kawasaki disease is a vasculitis disease with a highly activated immune system. Many studies have confirmed that T cells participate in the pathogenesis of KD and its IVIG treatment, but the type, proportion, CDR3 characteristics, and potential role of single/dual TCR T cells in KD disease and IVIG treatment are still unclear. In this study, the changes and potential effects of single/dual TCR T cells in KD patients before and after treatment were innovatively analyzed for the first time by scRNA-seq and TCR-seq technology. It was found that the proportion of dual TCR T lymphocytes increased significantly after IVIG treatment, and these T cells showed clonal expansion and a bias in V gene usage. Further dynamic analysis of dual TCR T cell subsets before and after IVIG treatment showed that dual TCR T lymphocytes, especially dual TCR CD8 T cells and Treg cells, play cruel roles in the pathology of KD and during IVIG treatment. This study not only further confirmed the immunomodulatory function of dual TCR T cells in diseases, but also provided strong support for clarifying the pathogenesis of KD and optimizing treatment strategies.

## Data Availability

The original contributions presented in the study are included in the article/**Supplementary Material**. Further inquiries can be directed to the corresponding author.
