# Supplementary material for: scRNA+TCR-seq reveals the pivotal role of dual receptor T lymphocytes in the pathogenesis of Kawasaki disease and during IVIG treatment
Source: Front Immunol. 2024 Oct 3;15:1457687. doi: 10.3389/fimmu.2024.1457687 (PMC11484261; doi:10.3389/fimmu.2024.1457687)
Supplement: Supplementary file 1 [file DataSheet1.docx]

**Supplementary Information**

**Supplementary table 1.**Detailed information on the clonal expansion of dual TCR T cells after treatment, as well as their ratio before and after treatment.

**Supplementary table 2.**TCR-seq details of 15 samples of single TCR T cells and dual TCR T cells.


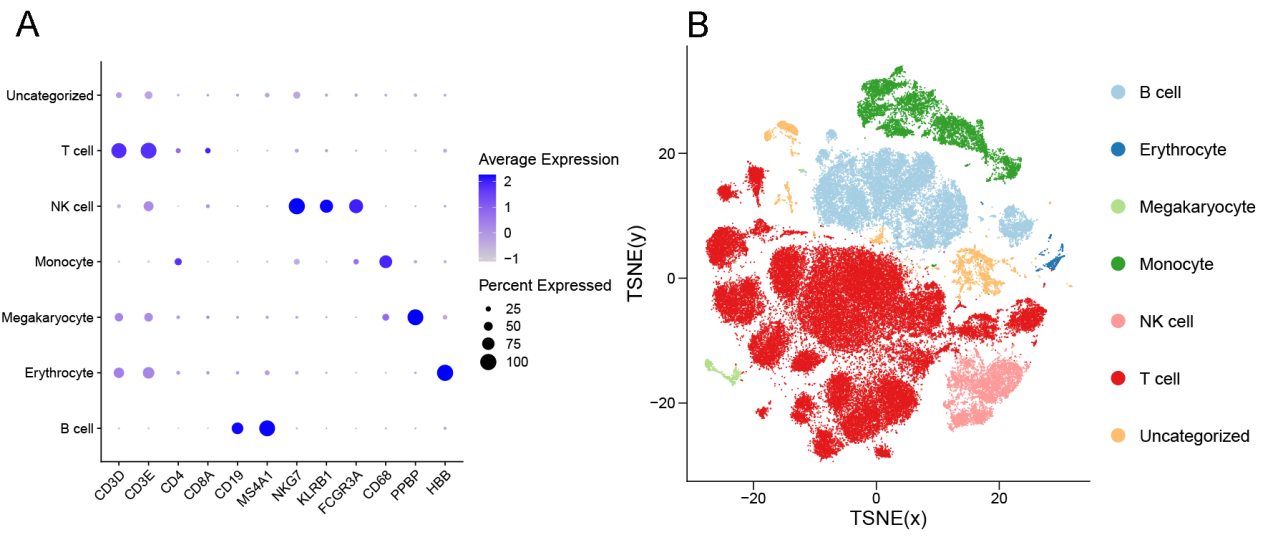


**Supplementary Figure 1.**The cell types and expression characteristics of PBMC samples were identified through scRNA-seq.


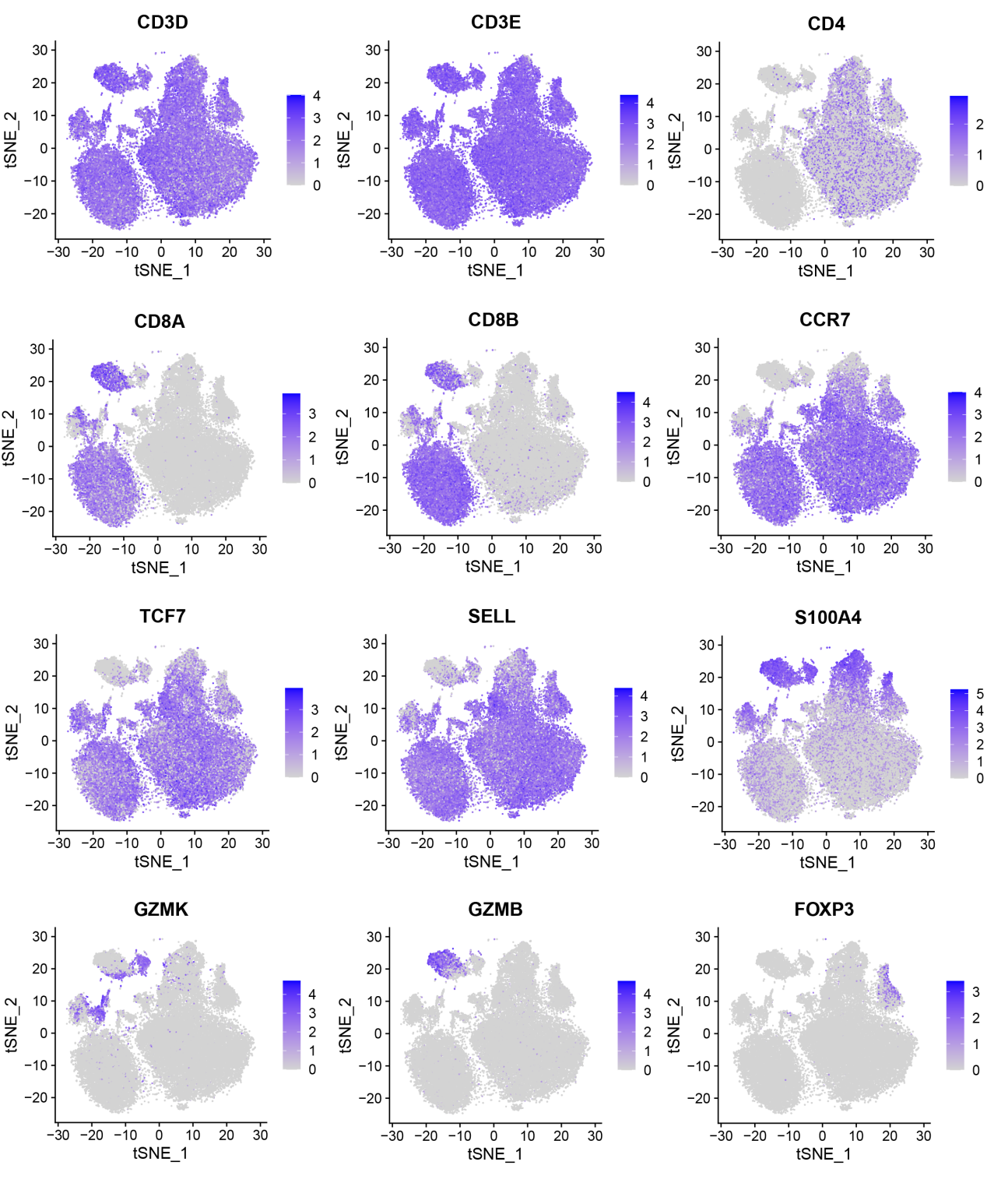


**Supplementary Figure 2.**Marker genes of T cell subsets.

**
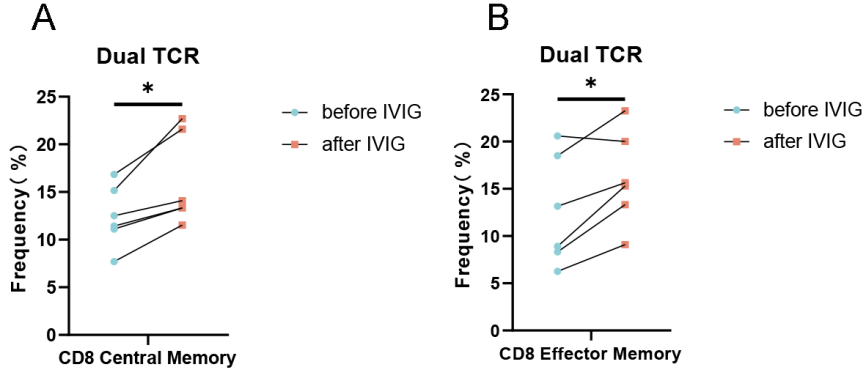
**

**Supplementary Figure 3.**Proportion of dual TCR CD8 central memory T cells and dual TCR CD8 effector memory T cells before and after treatment.


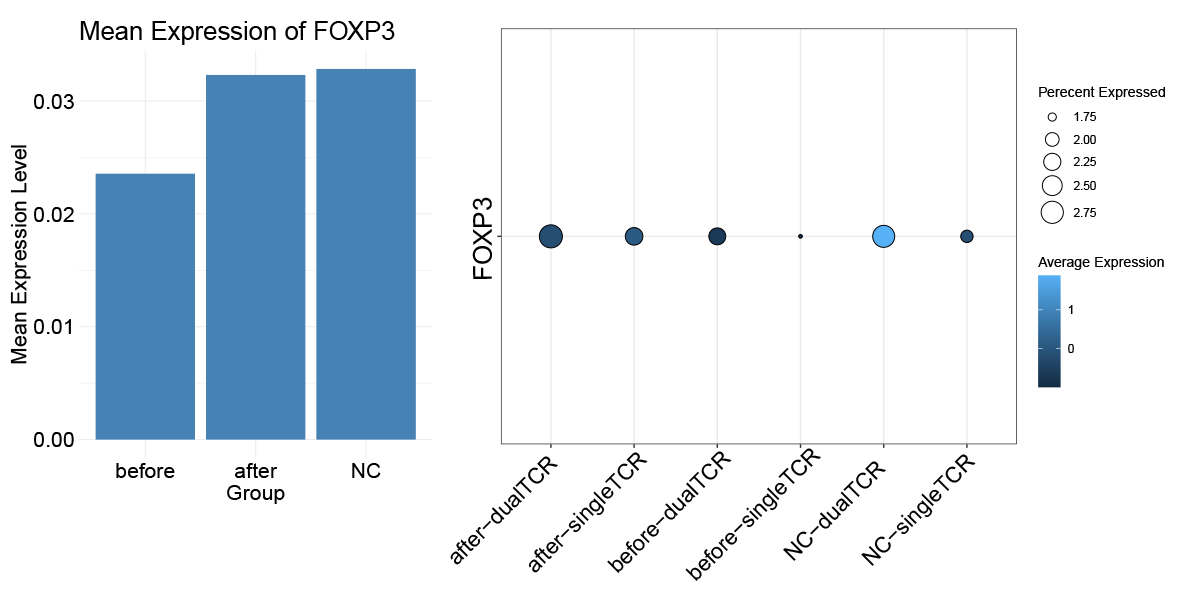


**Supplementary Figure 4.**Total *FOXP3* expression in three groups and *FOXP3* expression in single/dual TCR Treg cells among the three groups.
